# Information Encoding in Bursting Spiking Neural Network Modulated by Astrocytes

**DOI:** 10.3390/e25050745

**Published:** 2023-05-01

**Authors:** Sergey V. Stasenko, Victor B. Kazantsev

**Affiliations:** Laboratory of Advanced Methods for High-Dimensional Data Analysis, Lobachevsky State University of Nizhny Novgorod, 603022 Nizhny Novgorod, Russia

**Keywords:** spiking neural network, tripartite synapse, neuron, astrocyte

## Abstract

We investigated a mathematical model composed of a spiking neural network (SNN) interacting with astrocytes. We analysed how information content in the form of two-dimensional images can be represented by an SNN in the form of a spatiotemporal spiking pattern. The SNN includes excitatory and inhibitory neurons in some proportion, sustaining the excitation–inhibition balance of autonomous firing. The astrocytes accompanying each excitatory synapse provide a slow modulation of synaptic transmission strength. An information image was uploaded to the network in the form of excitatory stimulation pulses distributed in time reproducing the shape of the image. We found that astrocytic modulation prevented stimulation-induced SNN hyperexcitation and non-periodic bursting activity. Such homeostatic astrocytic regulation of neuronal activity makes it possible to restore the image supplied during stimulation and lost in the raster diagram of neuronal activity due to non-periodic neuronal firing. At a biological point, our model shows that astrocytes can act as an additional adaptive mechanism for regulating neural activity, which is crucial for sensory cortical representations.

## 1. Introduction

Mathematical and computational models in the form of so-called spiking neural networks (SNNs) have attracted growing attention from researchers in recent years [1,2,3]. In contrast to the classical formal neurons and artificial neuron networks (ANNs) widely used in many areas of computer science and information processing technologies, SNNs operate with a much more complex model of local neurons, synapses and network architectures. As more accurate brain circuit models, SNNs are expected to be much more powerful in solving non-trivial information processing tasks with associative or cognitive contents. However, there are crucial difficulties in training and tuning such networks to solve a specific task. It has become clear that these difficulties at the physical level are concerned with the complexity of SNN neurons and synapses. In particular, a small change in parameters (for example, when an input stimulus is given) can lead to the formation of complex dynamic modes that will not allow synaptic weights to be properly adjusted during training. The most common mode observed in biological neural networks is bursting [4]. Bursting underlies various processes associated with both information processing and neuropathologies [5,6], including epilepsy [7], and is being studied using dissociated cultures of brain neurons [4,8] and slice preparation. In particular, it has been shown that bursting can occur spontaneously [8,9] or in response to sensory input in the thalamus [10,11,12]. Thalamic neurons are prone to vacillating between burst and tonic firing modes in a state-dependent manner [13], which may serve as a dynamic gating mechanism for controlling the flow of information to the cortex [14,15,16,17,18,19]. However, it is unclear how the transition between burst and tonic firing modes is modulated in a dynamic sensory environment, coordinated across the neuronal population, and how this thalamic state transition affects information transmission. Sensory systems rapidly adapt to changes in stimulation to enhance information transmission in dynamic environments [20]. In the thalamocortical pathway, adaptation modulates sensory-evoked activity in thalamic neurons, impacting their downstream cortical targets. Thalamic adaptation desynchronizes firing activity, but the interaction with synchronized bursting across thalamic inputs to the cortex is not yet understood. This mechanism could robustly gate information flow based on bottom-up and top-down influences [11]. Bursting control mechanisms are being experimentally studied [21], but the issue remains relevant to this day [22]. One of the mechanisms controlling complex neuronal dynamics is short-term synaptic plasticity at timescales on the order of milliseconds [23,24,25]. In order to understand the control mechanisms of the network dynamics of entire brain structures, models have been proposed that imitate their activity and functions [26,27,28,29,30,31,32,33,34].

Finding ways to control and manage these modes has become an important task. One of the ways to control the dynamic modes, which also extends SNN’s standard capabilities, is to use an additional cell layer, in particular, astrocytes [35,36,37,38,39,40].

Astrocytes have been confirmed to participate in the synaptic transmission of information and regulate synaptic dynamics in neurobiological studies [41,42,43,44]. The inclusion of astrocytes in the classical “presynapse-postsynapse” scheme has led to the concept of a tripartite synapse, where astrocytes, through the calcium-dependent release of neuroactive chemicals such as gliotransmitters, can affect both the pre- and postsynaptic compartments of the synapse over a duration of seconds, which is relatively longer than the modulation of synaptic transmission by short-term synaptic plasticity.

In brief, the biochemical dynamics are as follows: when spikes are generated on a presynaptic neuron, neurotransmitters suh as glutamate are released from the presynaptic terminal. Some of these neurotransmitters may diffuse outside the synaptic cleft and bind to metabotropic glutamate receptors (mGluRs) on the astrocyte, which may be located close to the presynaptic terminal. This, in turn, triggers a chain of biochemical reactions, leading to the release of gliotransmitters (e.g., glutamate, adenosine triphosphate (ATP), D-serine, and GABA) from the astrocyte into the synaptic cleft and extrasynaptic space, subsequently modulating synaptic transmission (facilitation and depression) upon binding to pre- or postsynaptic receptors [45,46,47]. Recent studies have also shown that astrocytes are involved in the regulation of burst dynamics in cortical neurons in dissociated cultures [48]. More recently, it has also been found that astrocytes modulate thalamic sensory processing [49,50].

Many mathematical models have been proposed to understand the functional role of astrocytes in neuronal dynamics following experimental findings. One concept that emerged is the “dressed neuron”, which describes how astrocyte-mediated changes in neural excitability can impact neuronal function [51,52]. Several models have been proposed to explain the temporal dynamics of astrocytes, including the idea of astrocytes acting as frequency-selective “gate keepers” [53] and as regulators of presynaptic functions [54]. Experimental evidence has shown that gliotransmitters released by astrocytes can effectively modulate presynaptic facilitation and depression. Recently, the tripartite synapse model has been used to demonstrate how astrocytes participate in the coordination of neuronal network signaling, particularly in spike-timing-dependent plasticity (STDP) and learning, which are mechanisms responsible for neural synchrony and plasticity [55,56,57,58,59,60,61,62]. It is worth noting that the astrocytic modulation of neuronal activity has been modeled using biophysically detailed models and mean-field models, which describe observed experimental facts phenomenologically [63,64,65,66,67,68,69,70]. These models have revealed that functional gliotransmission is a complex phenomenon that depends on the nature of structural and functional coupling between astrocytic and synaptic elements. In the context of network dynamics, several models of spiking neural networks (SNNs) accompanied by astrocytes have been proposed, showing how astrocytes can enhance short-term memory performance by enabling the storage and recognition of highly overlapping information patterns [35,38,71,72]. In recent years, there have been experimental reports suggesting that astrocytes can influence the emergence of up–down synchronization in neuronal networks, but the underlying mechanisms are still uncertain. To explore how astrocytes can control this phenomenon, neural network models have been proposed, consisting of three populations of cells: excitatory neurons, inhibitory neurons, and astrocytes, interconnected by synaptic and gliotransmission events. These models demonstrate that the presence of astrocytes can promote the emergence of up–down regimes with realistic characteristics [73].

However, many aspects of information representation and processing in SNNs still remain open. In particular, sensory input may disturb the excitation–inhibition balance, causing spiking neural networks to oscillate between bursting and tonic firing modes, as observed in experimental studies on the thalamus. Experimental evidence suggests that astrocytes can act as an additional adaptive mechanism for regulating these bursting/tonic regimes, which is crucial in the formation of sensory cortical representations.

In this paper, we investigated the dynamics of a spiking neural network with tripartite synapses, modeled by a mean-field astrocyte model. We addressed the problem of sensory encodings with respect to the regulation of bursting and tonic firing modes in response to a sensory stimulus. We demonstrated that astrocytic modulation of neuronal activity can prevent stimulation-induced hyperexcitation and non-periodic bursting activity in SNNs. Such homeostatic regulation of neuronal activity enables the restoration of the image supplied during stimulation, which may be lost on the raster diagram of neuron activity due to non-periodic neuronal firing.

## 2. The Model

A spiking neural network consists of neurons modeled by a functional, biorelevant, and computationally efficient model of neurons proposed by Izhikevich [74], which can be described by the following equations:

(1) $$\left\{\begin{array}{c} \frac{dV_{i}}{dt}=0.04V_{i}^{2}+5V_{i}+140-U_{i}+I_{ext_{i}}+I_{syn_{i}}+I_{stim_{i}},\\ \frac{dU_{i}}{dt}=a\left(bV_{i}-U_{i}\right),\\ \mathrm{if}\;V_{i}\geq 30\;\mathrm{mV},\;\mathrm{then}\\ V_{i}=c,\\ U_{i}=U_{i}+d, \end{array}\right.$$

The parameters *a*, *b*, *c*, and *d* determine the type of neuron, $I_{ext_{i}}$ is the externally applied current, and the variables $V_{i}$ and $U_{i}$ describe the membrane potential and the process of activation and deactivation of potassium and sodium membrane channels, respectively. When the membrane potential $V_{i}$ reaches 30mV, the action potential is generated, and the variables are updated.

The parameters of the neuron model were chosen in such a way that the neuron was in an excitable mode, in which the generation of a spike impulse is initiated by some external influence (for example, an external stimulus or noise) [75]. This mode corresponds to the tonic spiking mode of the Izhikevich model [76]. We have fixed the parameters with the following values: a=0.02, b=0.5, c=−40mV, d=100, and $I_{ext}=40$.

$I_{syn_{i}}$ represents the sum of synaptic currents from all presynaptic neurons, *M*, with which this neuron is connected. In the model, synaptic currents were described as follows:

(2) $$I_{syn_{i}}=\sum_{j=1}^{M}y_{j,i}w_{j,i}.$$

In Equation (2), $w_{j,i}$ represents the weights of glutamatergic and GABAergic synapses between neurons, while *M* describes the count of presynaptic neurons that have actual connections with the *i*-th neuron. The weights of excitatory and inhibitory synapses are denoted by positive and negative signs, respectively. The variables $y_{j,i}$ represent the output signal (synaptic neurotransmitter) from the *i*-th neuron to the *j*-th neuron, which is involved in the generation of $I_{syn_{i}}$.

Initially, synaptic weights were randomly set for all connections within the range of 20 to 60. When a presynaptic neuron generates a spike, it results in a change in the concentration of the synaptic neurotransmitter $y_{j,i}$, which in turn leads to a change in the synaptic current on the postsynaptic neuron. The dynamics of $y_{j,i}$ can be described as follows:

(3) $$\frac{dy_{j,i}}{dt}=-\frac{y_{j,i}}{\tau_{y}}+b_{y}\theta \left(t-t_{sp}\right).$$

In Equation (3), $t_{sp}$ represents the time moments of consecutive presynaptic spikes, $\tau_{y}$ represents the relaxation time constant, and $b_{y}$ denotes the fraction of neurotransmitter release.

The generation of spikes in the neuron model leads to the release of a neurotransmitter. In our model, we specifically focus on the interaction of neurons with astrocytes, but only for glutamatergic synapses. This interaction has been extensively studied in previous modeling and experimental works, as it is considered an important mechanism for the formation of coherent neuronal excitation, as reported in [77,78]. In our network, GABAergic neurons play a crucial role in maintaining a balance between excitation and inhibition, preventing states of hyperexcitation.

To simplify the analysis, we adopt a phenomenological model to describe the dynamics of released glutamate. Using the mean-field approach, the mean extrasynaptic concentration of glutamate for each excitatory synapse, denoted by *X*, can be calculated using the following equation:

(4) $$\frac{dX_{e}}{dt}=-\frac{X_{e}}{\tau_{X}}+b_{X}\theta \left(t-t_{sp_{e}}\right).$$

The index *e* represents excitatory presynaptic neurons, and $b_{X}$ denotes the fraction of glutamate release, while $\tau_{X}$ is the relaxation time constant. When a spike is generated on the presynaptic neuron, a neurotransmitter is released, leading to an increase in the extrasynaptic neurotransmitter concentration due to diffusion processes. However, this concentration decreases over time with its characteristic time $\tau_{X}$. It is important to note that the mathematical descriptions of synaptic (Equation (3)) and extrasynaptic (Equation (4)) neurotransmitter dynamics are different, primarily due to the distinct time constants $\tau_{y}$ and $\tau_{X}$, respectively. The parameter values used in this model are $\tau_{X}=100$ ms and $\tau_{y}=4$ ms.

### 2.1. Astrocytic Dynamics

A portion of the extrasynaptic glutamate can bind to metabotropic glutamate receptors located on astrocyte. Subsequently, through a cascade of molecular transformations mediated by intracellular calcium elevation, the astrocyte releases gliotransmitters back into the extracellular space. However, for the purpose of our mathematical model, we have omitted a detailed description of these transformations and have only defined an input–output functional relationship between the neurotransmitter and gliotransmitter concentrations in the following form, as described in previous works [63,66,67]:

(5) $$\frac{dY_{j}}{dt}=-\alpha_{Y}Y_{j}+\frac{\beta_{Y}}{1+exp(-X_{e}+X_{thr})},$$

where e=1,2,3,… is the index of the excitatory neuron, *Y* represents the gliotransmitter concentration in the neighborhood of the corresponding excitatory synapse, and $\alpha_{Y}$ denotes the clearance rate. The parameter values used in our model are $\alpha_{Y}=120$ ms, $\beta_{Y}=0.5,$$X_{thr}=3.5$.

**Figure 1:**
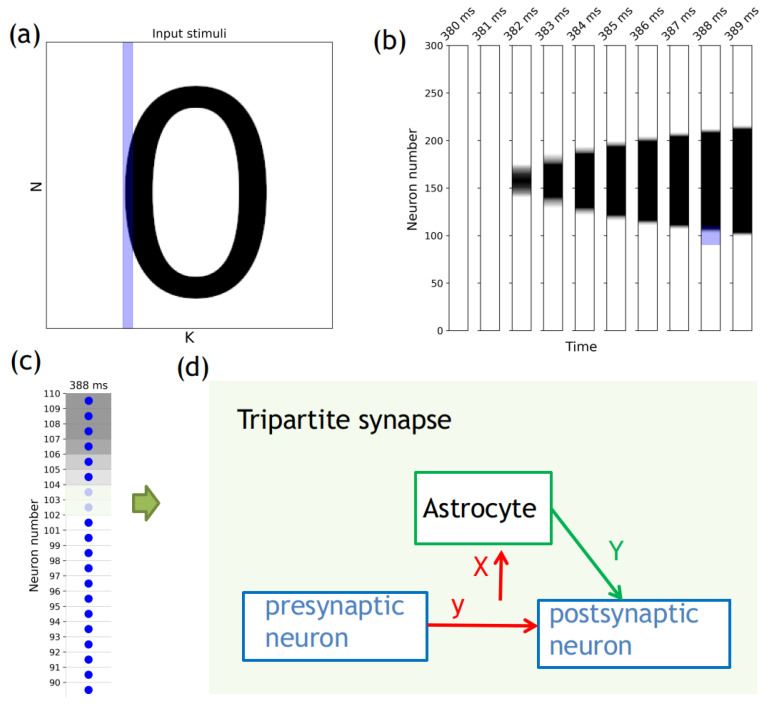
Scheme of the neuron–glial network and procedures for supplying an input stimulus to the neural network: (**a**) The input image is N by K (300×300 in our case), where N is the number of neurons and K is the duration of stimulus supply to the neural network; (**b**) The blue area from panel (**a**) divided into columns of pixel values supplied every subsequent ms; (**c**) The blue area from panel (**b**) shows blue dots representing neurons, and the color space around them represents pixel values ranging from 0 for white to $A_{S}$ for black; (**d**) The diagram of a tripartite synapse.

The second term in Equation (5) accounts for gliotransmitter production when the mean field concentration of gliotransmitters exceeds a certain threshold, $X_{thr}$. Please refer to Figure 1 for an illustration of the network construction and the neuron-to-astrocyte crosstalk specifically for excitatory glutamatergic synapses.

### 2.2. Astrocytic Modulation of Neural Activity

Based on experimental evidence, it has been observed that astrocytes can impact the probability of neurotransmitter release, resulting in either potentiation or depression of synaptic transmission [79,80,81]. This, in turn, leads to the modulation of synaptic currents. In our model, we specifically incorporate depression of synaptic transmission, which is manifested as a decrease in the probability of neurotransmitter release, for glutamatergic synapses. The mathematical representation of this depression is as follows:

(6) $$I_{syn_{i}}=\sum_{j=1}^{M}y_{j,i}w_{j,i}\left(1-\frac{\gamma_{Y}}{1+exp(-Y_{j}+Y_{thr})}\right).$$

In our model, the synaptic current in the postsynaptic neuron, denoted as $I_{syn_{i}}$, is the sum of all synaptic currents from presynaptic neurons. The weight of the glutamatergic synapses between neurons is represented by $w_{j,i}$, and the coefficient of astrocyte influence on synaptic connection is denoted as $\gamma_{Y}$. For illustrative purposes, we fix the threshold of gliotransmitter concentration that triggers astrocyte influence on synaptic connection at $Y_{thr}=2$.

In our current study, we have made the assumption that astrocytes modulate only the excitatory neurons, while the inhibitory neurons function in the classical pre- to postsynaptic information transmission role.

### 2.3. Stimulation Current

A set of input images of numbers from 0 to 9 was prepared using a freely distributed raster graphics editor (GIMP [82]). The image itself was 300 by 300 pixels (denoted as N×K from Figure 1a). The image was encoded from 0 to 1, $S_{i}$, where zeros represented pixels without color, and units were were represented by black. Hence, the equation for the stimulation current, $I_{stim_{i}}$, can be written in the following form:

(7) $$I_{stim_{i}}=S_{i}\times A_{S},$$

where $A_{S}$ represents the amplitude of the stimulus.

Each column of the stimulation current matrix was applied to each neuron in the network for 1 ms. The total duration of the image feed was determined by the number of columns in the matrix, which corresponds to the width of the input image (300 ms).

### 2.4. Neural Network

The model consists of a single-layer spiking neural network comprising excitatory and inhibitory neurons in a ratio of 4 to 1. This ratio is consistent with experimental studies that have shown the ratio of excitatory to inhibitory neurons to fall within a relatively narrow range of 3:1 to 9:1, with inhibitory neurons comprising 10–25% of the total neuronal population [83,84,85,86,87,88]. Neurons are connected in an “all-to-all” manner, with a probability of connection set at 5% for excitatory synapses and 20% for inhibitory synapses [89].

The number of neurons in the neural network was determined by one of the dimensions (N = 300) of the image fed to the network (Figure 1a). Another dimension (K = 300) of the supplied image determined the time of supplying the image pixel columns to the neural network. The white color in Figure 1a–c indicates pixel values equal to 0. Figure 1b shows a breakdown of the blue region of the input image (Figure 1a) in the space of neurons and the time of the stimulus. Figure 1c shows a part of the neural network, which at a time of 388 ms is supplied with a part of the stimulus marked by the blue area in Figure 1b. Figure 1d shows a diagram of a tripartite glutamatergic synapse between neurons #102 and #103 from Figure 1c. Spike generation on a presynaptic neuron results in the release of a neurotransmitter (glutamate) into the synaptic cleft. Part of the released neurotransmitter reaches the astrocyte and, by binding to metabotropic receptors [90], activates it, resulting in the release of the gliotransmitter (glutamate). The gliotransmitter reaches the receptors of the postsynaptic neuron and leads to a depression of synaptic transmission. Our model is built using a mean-field approach for neurotransmitters and gliotransmitters, focusing on the dynamics of synapses while taking into account these regulations.

### 2.5. Numerical Simulation Method

The Euler method with a step size of 0.01 ms was employed for numerical integration. For implementing numerical methods and data analysis, the Python programming language [91] was used, along with the NumPy library for arrays, the Pandas library for data processing and analysis, Brian2 [92] for model simulation, and the Matplotlib and Seaborn [93] libraries for data visualization and analysis.

### 2.6. Image Similarity Metrics

To compare the original stimulus applied to the neural network with its representation in the form of spike activity, we utilized several classical metrics [94] that are commonly used due to their interpretability and universality, including the mean squared error (MSE), root-mean-squared error (RMSE), peak signal-to-noise ratio (PSNR), and structural similarity index (SSIM). Below is a brief description of each metric.

The mean squared error (MSE [94,95,96]) is the most common and traditional measure of similarity between two images. It calculates the squared error between estimated values (comparison image, $\hat{g}\left(n,m\right)$) and actual values (original image, g(n,m)) of pixels, according to the following equation:

(8) $$MSE=\frac{1}{MN}\sum_{n=0}^{M}\sum_{m=1}^{N}{\left[\hat{g}\left(n,m\right)-g\left(n,m\right)\right]}^{2}$$

The resulting values are not standardized and can therefore be quite large. An MSE value of zero indicates that the images are absolutely similar.

The root-mean-squared error (RMSE [94]) overcomes one of the disadvantages of the MSE metric, which is the issue of obtaining large values. It is commonly used to measure the difference between the predicted value and the actual value by estimating the magnitude of the error, and can be described by the following equation:

(9) $$RMSE\left(\hat{\theta }\right)=\sqrt{MSE(\hat{\theta })},$$

where $\hat{\theta }$ is estimated with respect to a given estimate parameter θ.

Since a noise signal, $I_{ext_{i}}$, is also fed to the neural network, we also used the PSNR metric (peak signal-to-noise ratio) [94,97] to compare images. This metric allows us to assess the relationship between the maximum possible signal power and the power of the distorting noise that affects the reliability of its presentation. It can be calculated as follows:

(10) $$PSNR=10\log_{10}\left(peakval^{2}\right)/MSE$$

PSNR is a variant of MSE and is used for pixel-by-pixel comparison. The higher the PSNR value, the better the quality of the compared image will be.

Since the input stimulus, $I_{stim_{i}}$, is divided into parts and delivered in parts during the calculation of the model for a given time, structural changes in the resulting pattern can be observed when the stimulus is represented by neural network spike activity. To assess its similarity with the original stimulus, we used the structural similarity index (SSIM) method [94], which is calculated as follows:

(11) $$SSIM\left(x,y\right)=\frac{\left(2\mu_{x}\mu_{y}+c_{1}\right)\left(2\sigma_{x,y}+C_{2}\right)}{\left(\mu_{x}^{2}+\mu_{y}^{2}+C_{1}\right)\left(\sigma_{x}^{2}+\sigma_{y}^{2}+c_{2}\right)},$$

where $\mu_{x}$ is the average value for the first image, $\mu_{y}$ is the average value for the second image, $\sigma_{x}$ and $\sigma_{y}$ are the standard deviations for the first and second image, respectively, $C_{1}$ and $C_{2}$ are correction factors, and $\sigma_{x,y}$ is the covariance, which is calculated as follows:

(12) $$\sigma_{x,y}=\mu_{x,y}-\mu_{x}\mu_{y}$$

The SSIM is related to the quality and perception of the human visual system (HVS color model). In this metric, instead of using traditional error summation methods, SSIM models image distortion as a combination of three factors: correlation loss, brightness distortion, and contrast distortion. The peculiarity is that it always lies in the range from −1 to 1, and when its value is equal to 1, it means that we have two identical images. It has also been shown [98] that, unlike SSIM, MSE and PSNR do a poor job of recognizing structural content in images, since different types of degradation applied to the same image can give the same MSE value. To calculate these metrics, the Sewar library [99] was used.

## 3. Results

**Figure 2:**
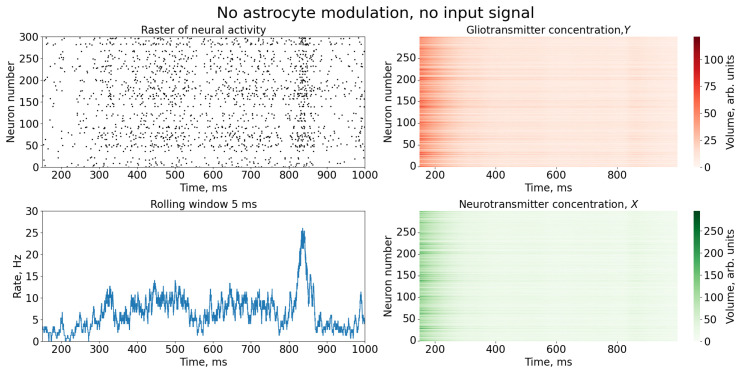
Time series of neural activity (**upper left** figure in the form of a raster diagram); the corresponding rate of activity of the neural network is the sum of the spikes of the neurons of the network, calculated in a time window 5 ms (**lower left** figure) and time series of concentrations of gliotransmitters, *Y*, (**upper right** figure) and concentrations of neurotransmitters, *X* (**lower right** figure).

We started simulations of Equations (1)–(8) from the autonomous dynamics of SNN without the astrocyte action and external stimulation. Due to the presence of the inhibitory neurons, network firing was balanced, preventing hyperexcitation. The initial high-frequency burst that occurs due to an arbitrary initial conditions is followed by rare spiking dynamics, as illustrated in Figure 2. The SNN was untrained, and hence, its spikes occur in an irregular manner due to an uncorrelated subthreshold noise drive.

**Figure 3:**
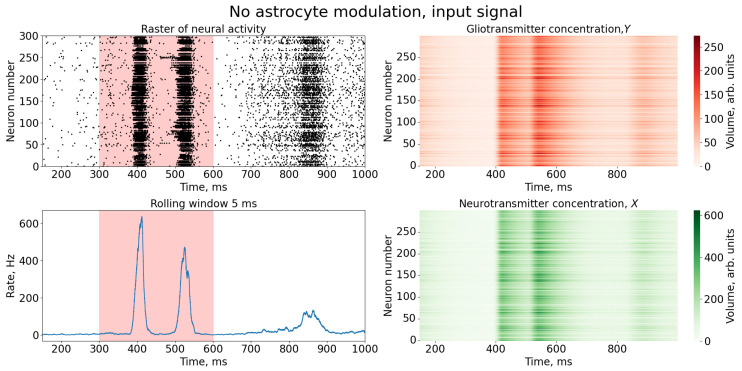
Time series of neural activity (**upper left** figure in the form of a raster diagram); the corresponding rate of activity of the neural network is the sum of the spikes of the neurons of the network, calculated in a time window of 5 ms (**lower left** figure) and time series of concentrations of gliotransmitters, *Y*, (**upper right** figure) and concentrations of neurotransmitters, *X* (**lower right** figure). The red area in the Figure indicates the time duration of the input stimulus.

Next, we stimulated the SNN by feeding the image as the number 0 (Figure 1a) according to the Equation (7) for 300 ms. The space-time dynamics of the SNN is illustrated in Figure 3. Note that the feedback from the astrocyte layer given by Equation (6) has not been activated yet. Neurotransmitter and gliotransmitter concentrations simply followed the average rate of spiking in the corresponding SNN’s sites. As the stimulation was excitatory, the SNN responded by transitioning to its hyperexcitation mode, displaying bursting dynamics after the stimulation. Bursts were characterized by quasi-synchronous population discharges with a significantly high spiking rate. Note that, in this case, all features of the spatiotemporal pattern (Figure 3) were diffused by the hyperexcitation dynamics. Additionally, due to the stimulation current also containing zero values (areas of the pattern without color), the beginning of stimulation did not coincide with the occurrence of neuronal synchronization and the formation of a burst.

**Figure 4:**
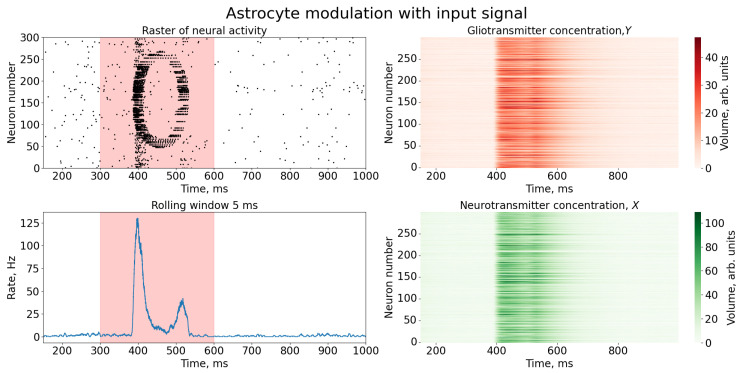
Time series of neural activity (**upper left** figure in the form of a raster diagram); the corresponding rate of activity of the neural network is the sum of the spikes of the neurons of the network, calculated in a time window of 5 ms (**lower left** figure) and time series of concentrations of gliotransmitters, *Y*, (**upper right** figure) and concentrations of neurotransmitters, *X* (**lower right** figure). The red area in the Figure indicates the time duration of the input stimulus.

**Figure 5:**
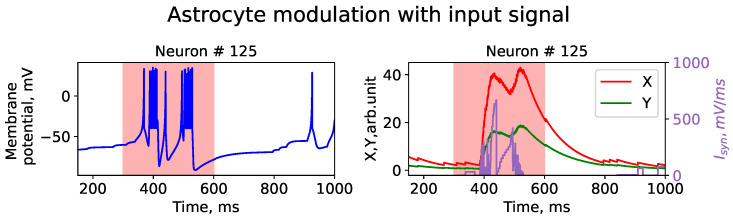
Time series of membrane potential changes, *V*, for single neuron (**left** figure) and time series of gliotransmitter concentrations, *Y*, (**right** figure), neurotransmitter concentrations, *X*, (**right** figure), and synaptic current, $I_{sin}$. The red area in the figure indicates the timing of the input stimulus.

Figure 4 depicts the results of astrocytic feedback leading to synaptic plasticity depression. The dynamics of an individual synapse and a typical SNN neuron are shown in Figure 5. The raster plot suggests the presence of an image-like pattern in the spike dynamics. It is noteworthy that the quasi-synchronous bursts were eventually suppressed, and the SNN exhibited rare bursts, indicating a return to “normal dynamics”. This was achieved through astrocytic modulation of neurotransmitter release probability and corresponding processes of inhibition and excitation. Specifically, the parameters of astrocyte-mediated feedback were fine-tuned such that the high-frequency bursts, activated when the image nucleus approached the entrance, induced astrocyte feedback, leading to the suppression of excitatory synaptic transmission. Subsequently, after the core of the image passed through the front door, the network quickly returned to a rare peak mode, displaying the shape of the image on the raster diagram.

**Figure 6:**
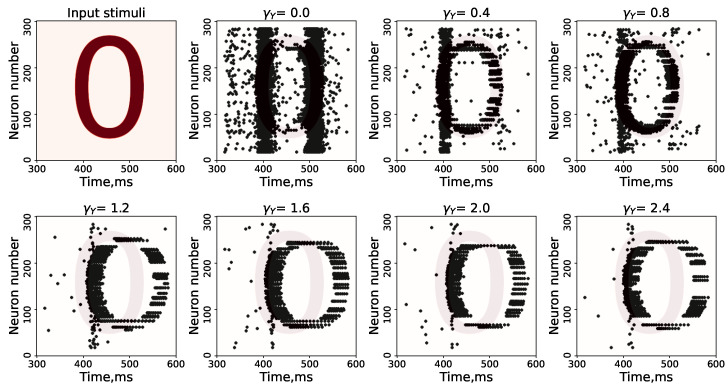
Part of the raster diagram of neural activity, demonstrating the representation of the supplied pattern at different parameter values of $\gamma_{Y}$.

Next, we analyzed how the strength of the astrocyte feedback, controlled by the parameter $\gamma_{Y}$ (Figure 6), affected the quality of the SNN image representation. With the parameter $\gamma_{Y}$ regulating the astrocytic depression of synaptic transmission, the image of the number 0 begins to appear and to disappear as non-periodic burst activity, which prevents the image from being represented on the SNN. Note that starting from a value of the parameter, $\gamma_{Y}$, equal to 0.8, the image begins to be distorted due to over-depression of the synaptic transmission dynamics but remains visually recognizable.

**Figure 7:**
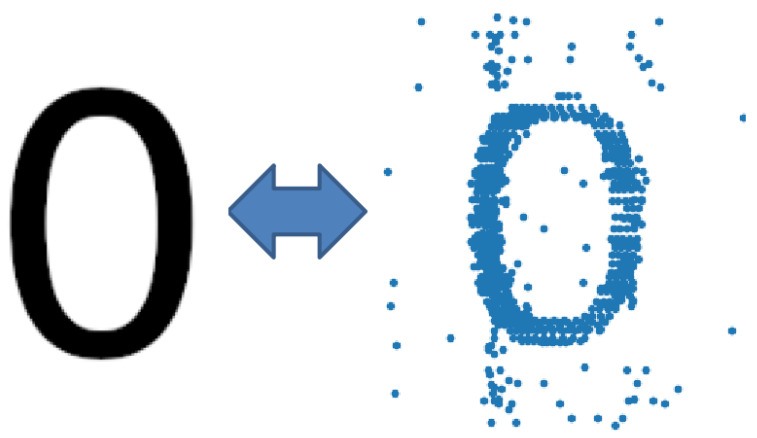
Image comparison scheme. On the left, the original stimulus image that is fed to the spiking neural network. The right picture shows a blurred scan of the image that is restored by spiking neural network.

Next, we made a qualitative comparison of the original simulation fed to the spiking neural network in the form of the number 0 and the image restored by the spiking neural network on a raster diagram of neural activity. An example of the comparison scheme is shown in Figure 7.

**Figure 8:**
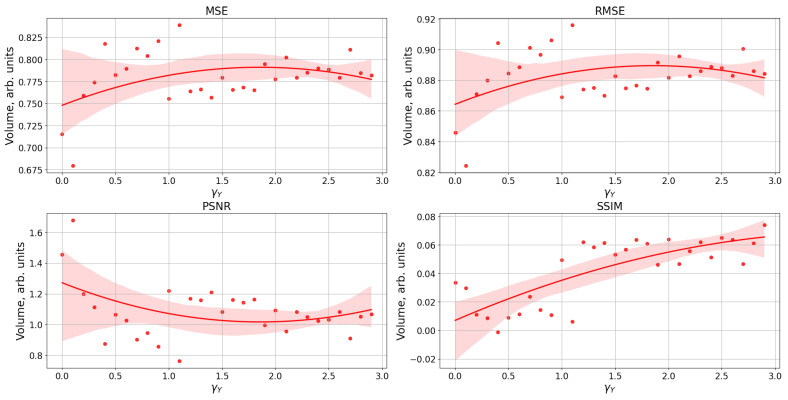
The dependence of the image comparison metrics (MSE, RMSE, PSNR, and SSIM) for comparing the applied stimulus and the reproduced pattern by the neural network on the parameter value $\gamma_{Y}$. MSE—mean square error. RMSE—root-mean-square error. PSNR—peak signal-to-noise ratio. SSIM—structural similarity index method.

Figure 8 shows a comparison of a raster plot of a spiking neural network with a reproduced image of the number 0 with the image fed to the neural network when changing the astrocytic regulation of synaptic transmission ($\gamma_{Y}$) using various metrics from the field of computer vision and machine learning used for image comparison tasks: MSE, RMSE, PSNR, and SSIM [94].

As the parameter $\gamma_{Y}$ (astrocytic synaptic depression) increases, the deformation of the network activity pattern reproducing the stimulus input leads to poor performance of image comparison metrics (MSE, RMSE, and PSNR) based on pixel-by-pixel calculation of the MSE error, as shown in Figure 8. However, the image remains recognizable and distinguishable in the pattern of network activity, as shown in Figure 6.

This can be explained by two reasons. Firstly, the image was fed to the spiking neural network partially within 300 ms, meaning that only a part of the image was supplied within 1 ms. Secondly, the image was restored in the spiking neural network operating in its bursting mode, which resulted in changes in synaptic weights and shifts in the image. These metrics (MSE, RMSE, and PSNR) used for pixel-by-pixel comparison may not be suitable for comparing images that are semantically close but differ in pixel-level details. Therefore, we can conclude that these metrics may not be optimal for evaluating images with subtle semantic differences.

At the same time, the SSIM metric demonstrates an improvement in the similarity between the original image of the input stimulus and the network activity pattern as the parameter increases. This reflects the fact that the image was deformed relative to the pixel space but remained structurally similar to the input stimulus.

Additional results obtained using other input images are illustrated in Appendix A and in the Supplementary Materials.

### Study of Neuron and Neural Network Parameters

Next, the parameters of the neurons (refractory period) and of the neural network (ratio of excitatory/inhibitory neurons and connection probability between neurons) were studied for the representation of the input image in the form of a pattern of neural activity, without and with astrocyte modulation.

**Figure 9:**
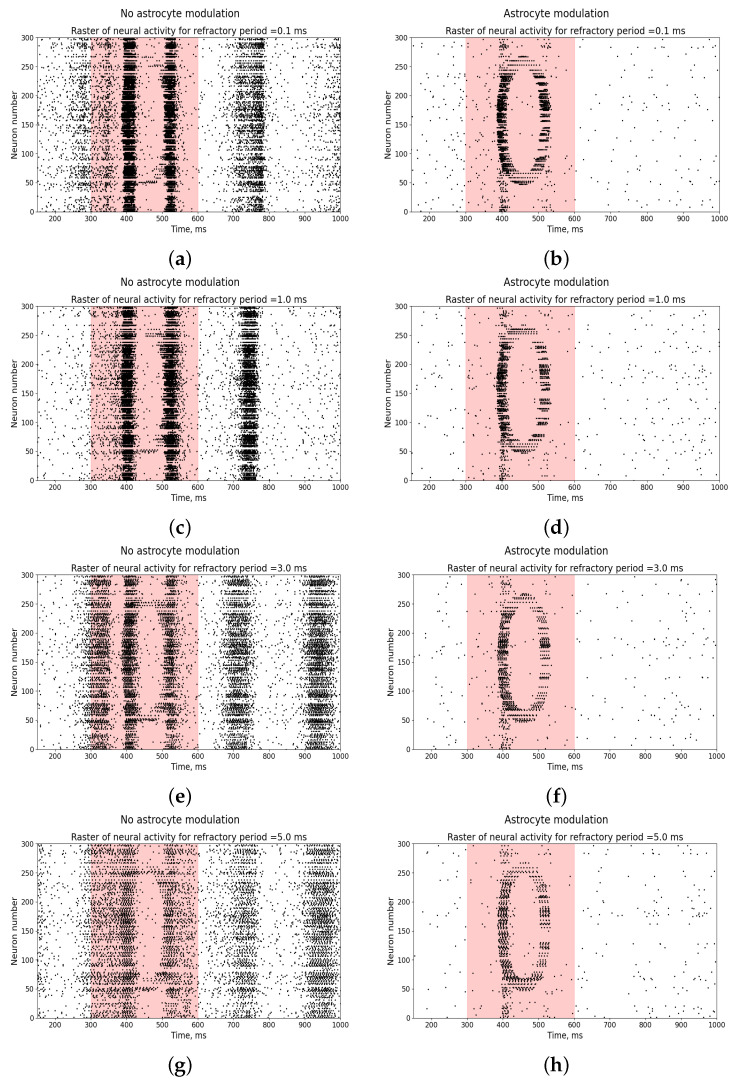
Raster diagram of neural activity when an input stimulus from Figure 1a is applied to the neural network (red area) in the presence (right panel—(**b**,**d**,**f**,**h**)) and absence (left panel—(**a**,**c**,**e**,**g**)) of astrocytic modulation under different parameters of the neuron refractory period: 0.1 ms, 1 ms, 3 ms, and 5 ms.

A change in the refractory period of a neuron does not lead to the disappearance of burst activity and the manifestation of a pattern of neuronal activity reproducing the input stimulus (left panel in the Figure 9). At the same time, the inclusion of astrocytic modulation in such a network leads to the disappearance of burst activity and the manifestation of the input stimulus in the form of a pattern of neural activity (panel on the right in the Figure 9).

Next, we investigated the effects of the ratio of excitatory and inhibitory neurons in the network on the reproduction of the input stimulus in the form of a pattern of neural activity in the presence and absence of astrocytic modulation.

**Figure 10:**
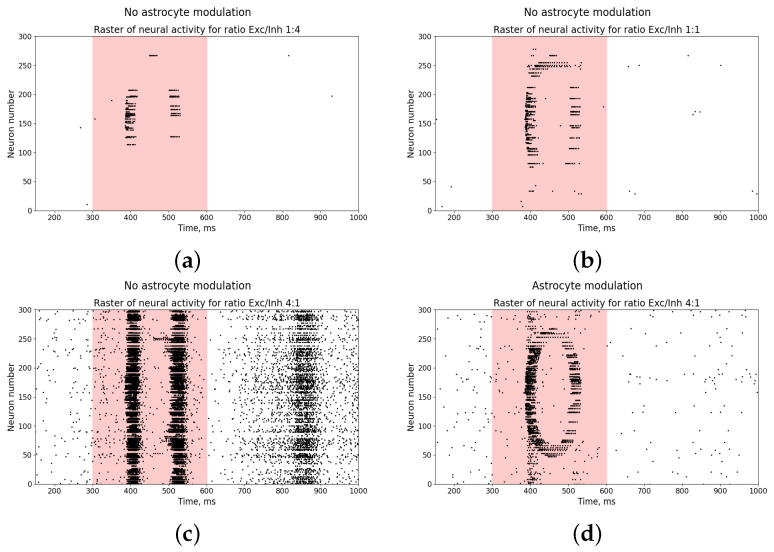
Raster diagram of neural activity when an input stimulus from Figure 1a is applied to the neural network (red area) in the presence (**d**) and absence (**a**–**c**) of astrocytic modulation at different ratios of excitatory and inhibitory neurons: 1:1, 1:4, and 4:1.

As can be seen, the input stimulus almost does not appear as a neural network activity pattern with an equal or larger number of inhibitory neurons (Figure 10a,b). However, such a ratio of neurons is not experimentally observable [83,84,85,86,87,88], in contrast to the case shown in Figure 10c. When the percentage of excitatory neurons is exceeded (Figure 10c), the neural network demonstrates the appearance of burst dynamics in response to the input stimulus (Figure reffig:schemea), and the manifestation of an activity pattern, as well as the removal of burst dynamics, is possible only in the presence of astrocytic modulation (Figure 10c).

**Figure 11:**
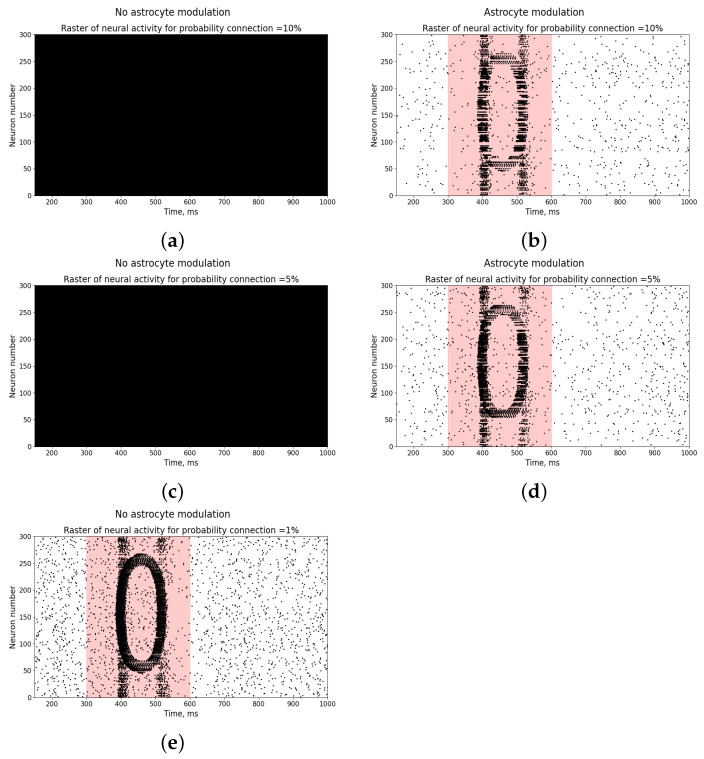
Raster diagram of neural activity when an input stimulus from Figure 1a is applied to the neural network (red area) in the presence (**b**,**d**) and absence (**a**,**c**,**e**) of astrocytic modulation at different connection probabilities between neurons: 1%, 5%, and 10%.

The last case pertains to the study of the influence of the probability of connection between neurons on the representation of the input stimulus as a pattern of neural activity in the absence and presence of astrocytic modulation. As can be seen, only with a very low probability of connection (1%) is it possible to represent the input stimulus without the participation of astrocytes (Figure 11e). However, with an increase in the probability of connection to 5% or more, hyperexcitation of the neural network occurs in response to the input stimulus (Figure 11a,c), which requires astrocytic modulation to correct it and represent the input stimulus as a pattern of neural activity (Figure 11b,d).

## 4. Discussion

The growing interest in the possible role of astrocytes in the regulation of neural activity and various processes in the brain has led to the emergence of a branch of computational neuroscience called *computational glioscience* [100]. Similar to recent modeling papers on neuron–astrocyte networks (see, in particular, [38,40]), our study also demonstrates that astrocytes may play a crucial role not only in maintaining the spiking dynamics of spiking neural networks but also in the implementation of an interface between information patterns and their internal representation in neuronal circuits.

In our spiking neural network model, synaptic weights at the initial moment of time were determined randomly and subsequently corrected by astrocytes. The observed non-periodic synchronous activity upon presentation of the stimulus was verified for most images, indicating the destruction of synchronization during astrocytic regulations.

On the one hand, our model is a drastic simplification of the cognitive processing that occur in the real brain. However, on the other hand, it is expected to be highly predictive because it incorporates facts observed at the molecular–cellular level in experiments [42,45,46,90].

We also note that the model considers a specific modulation of the spiking neural network, in which neurons could become synchronized and exhibit non-periodic bursting dynamics as a consequence of hyperexcitation. To suppress the hyperexcitability of neurons, depression of synaptic transmission was introduced into the model using astrocytic regulation of neuronal activity. In the proposed model, we focused on the consideration of one of the basic functions of astrocytes observed in the glutamatergic synapse. Further complexity of the model could be achieved by introducing astrocytic regulation in the GABAergic synapse, as well as bidirectional astrocytic regulation, may yield possible ways to control the quality of spiking image representation in SNN-based information processing.

To date, various neuromorphic devices based on memristors are being implemented, which allow for the imitation of basic regulatory mechanisms, including those of astrocytes [101]. Understanding the control mechanisms of the complex dynamics of neural networks at the level of synapse dynamics due to astrocytic modulation (which occurs over longer timescales than short-term synaptic plasticity) will enable their incorporation into new memristive devices and expand the possibilities and systems of neuromorphic computing.

## 5. Conclusions

We have investigated the performance of an SNN-plus-astrocyte computational model in processing space-time external information patterns. By feeding images of numbers from 0 to 9 as a spatiotemporal signal, we have shown that the spiking neural network can generate non-periodic neuronal firing, which leads to the “loss” of the image on the raster diagram of neural activity. Astrocytic regulation of neuronal activity by suppressing synaptic transmission makes it possible to “restore” the image on a raster diagram of neuronal activity. However, qualitative pixel-by-pixel measurements (using quality indicators such as MSE, RMSE, and PSNR) of the original image supplied as a stimulus and the image displayed on the raster diagram of neuronal activity with increasing astrocytic depression of synaptic transmission show differences, although the SSIM structural similarity metric shows an improvement in the representation of the input signal by the neural network. Changes in the feedback of astrocytes led to blurring of the image core, which occurred mainly due to SNN hyperexcitation upon stimulus delivery and strengthening of excitatory synaptic connections. Moreover, the study of various parameters (such as the neuron refractory period, ratio of excitatory and inhibitory neurons, connection probability between neurons) of the neural network showed the significance of astrocytic modulation, regardless of the architecture of the network. Astrocytes, operating at a much slower time scale, provided an “inertial” buffer to prevent hyperexcitation of firing neurons. Our work demonstrates that astrocytes can serve as an adaptive mechanism for regulating neuronal activity between bursting and tonic modes in response to sensory input. This is crucial in the formation of sensory cortical representations.

## Figures and Tables

**Figure A1:**
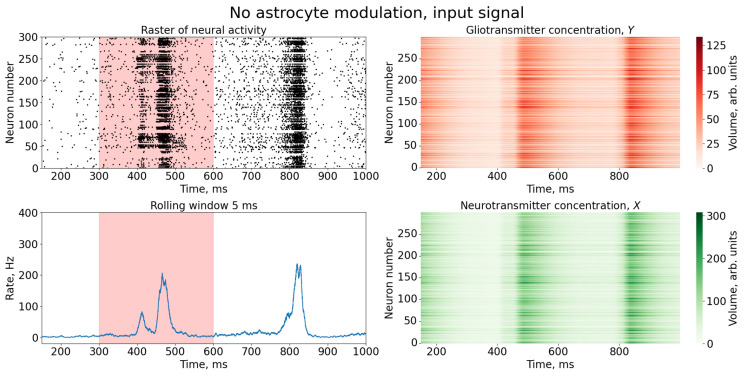
Time series of neural activity (**upper left** figure in the form of a raster diagram); the corresponding rate of activity of the neural network is the sum of the spikes of the neurons of the network, calculated on a time window of 5 ms (**lower left** figure) and time series of concentrations of gliotransmitters, *Y* (**upper right** figure) and concentrations of neurotransmitters, *X* (**lower right** figure). The red area in the Figure indicates the time duration of the input stimulus.

**Figure A2:**
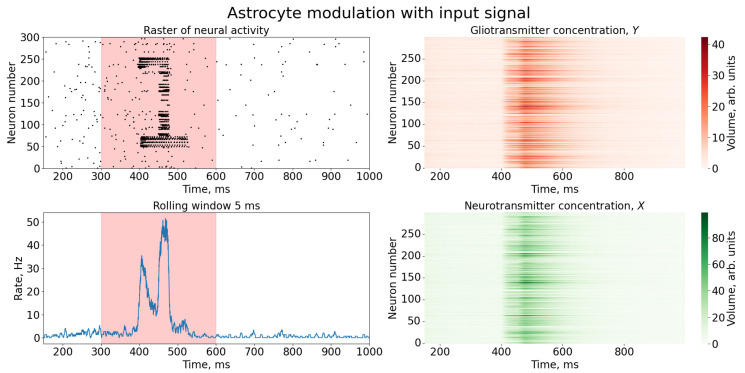
Time series of neural activity (**upper left** figure in the form of a raster diagram); the corresponding rate of activity of the neural network is the sum of the spikes of the neurons of the network, calculated on a time window of 5 ms (**lower left** figure) and time series of concentrations of gliotransmitters, *Y* (**upper right** figure), and concentrations of neurotransmitters, *X*, (**lower right** figure). The red area in the Figure indicates the time duration of the input stimulus.

**Figure A3:**
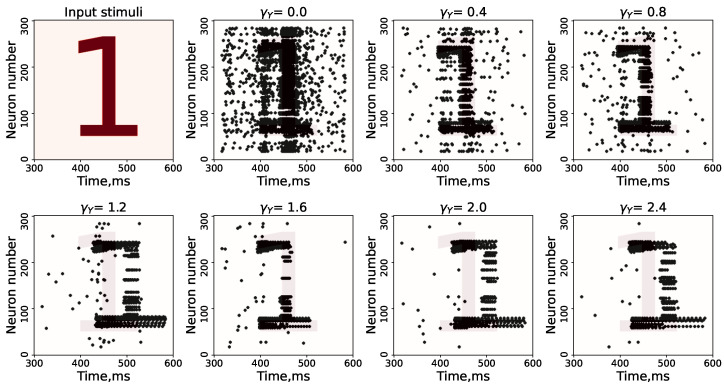
Part of the raster diagram of neural activity demonstrating the representation of the supplied pattern at different parameter values $\gamma_{Y}$.

**Figure A4:**
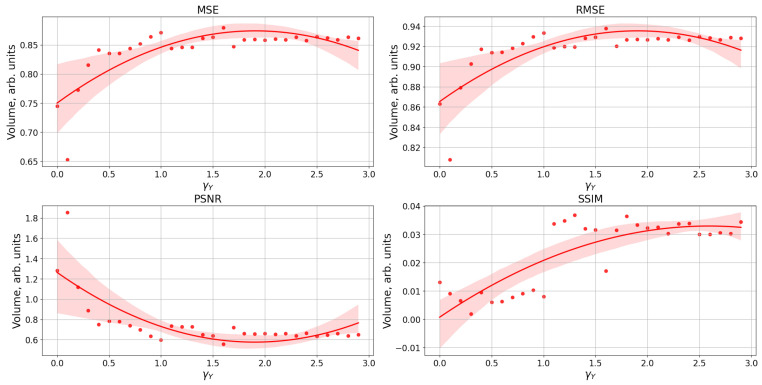
The dependence of the image comparison metrics (MSE, RMSE, PSNR, and SSIM) for comparing the applied stimulus and the reproduced pattern by the neural network on the parameter value $\gamma_{Y}$. MSE—mean square error. RMSE—root-mean-square error. PSNR—peak signal-to-noise ratio. SSIM—structural similarity index method.

**Figure A5:**
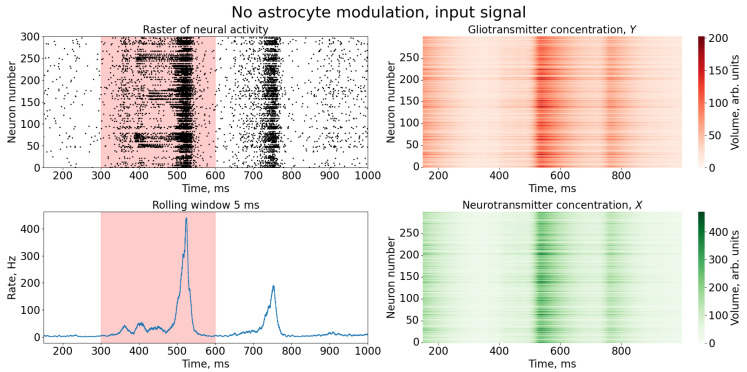
Time series of neural activity (**upper left** figure in the form of a raster diagram), the corresponding rate of activity of the neural network is the sum of the spikes of the neurons of the network, calculated on a time window of 5 ms (**lower left** figure) and time series of concentrations gliotransmitters, *Y* (**upper right** figure), and concentration neurotransmitters, *X* (**lower right** figure). The red area in the Figure indicates the time duration of the input stimulus.

**Figure A6:**
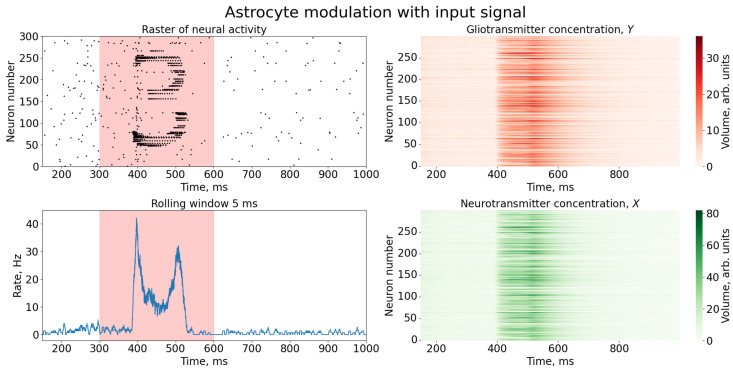
Time series of neural activity (**upper left** figure in the form of a raster diagram); the corresponding rate of activity of the neural network is the sum of the spikes of the neurons of the network, calculated on a time window of 5 ms (**lower left** figure) and time series of concentrations of gliotransmitters, *Y* (**upper right** figure), and concentrations of neurotransmitters, *X* (**lower right** figure). The red area in the Figure indicates the time duration of the input stimulus.

**Figure A7:**
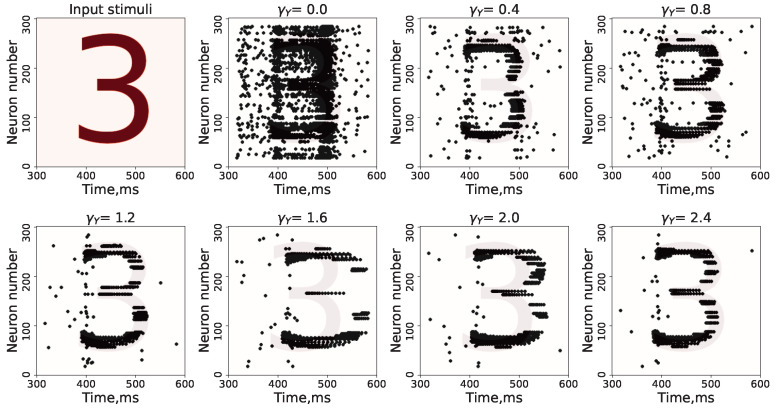
Part of the raster diagram of neural activity demonstrating the representation of the supplied pattern at different parameter values $\gamma_{Y}$.

**Figure A8:**
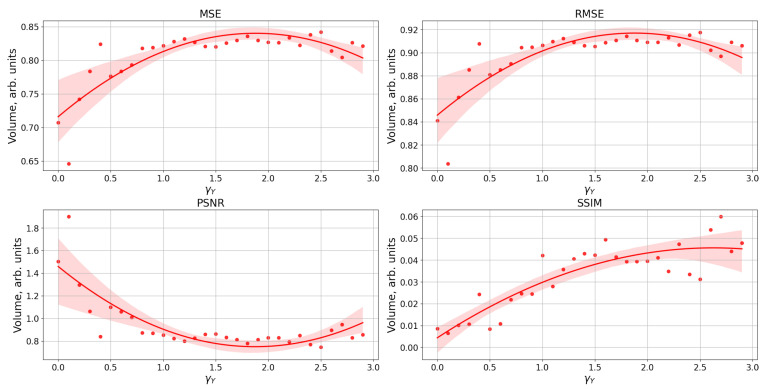
The dependence of the image comparison metrics (MSE, RMSE, PSNR, and SSIM) for comparing the applied stimulus and the reproduced pattern by the neural network on the parameter value $\gamma_{Y}$. MSE—mean square error. RMSE—root-mean-square error. PSNR—peak signal-to-noise ratio. SSIM—structural similarity index method.

## Data Availability

The data that support the findings of this study are available from the corresponding author upon reasonable request. The code used to produce the results presented herein is available in a public GitHub repository at https://github.com/sstasenko/burstingSNN (accessed on 20 February 2023).

## Supplementary Materials

The following supporting information can be downloaded at: https://www.mdpi.com/article/10.3390/e25050745/s1.
