# Supplementary material for: Information Encoding in Bursting Spiking Neural Network Modulated by Astrocytes
Source: Entropy (Basel). 2023 May 1;25(5):745. doi: 10.3390/e25050745 (PMC10217185; doi:10.3390/e25050745)
Supplement: Supplementary file 1 [file entropy-25-00745-s001.zip › entropy-2282406-supplementary.pdf]

Supplementary Materials: Information encoding in bursting spiking neural network modulated by astrocytes

Sergey V. Stasenko [1]*, and Victor B. Kazantsev [1]

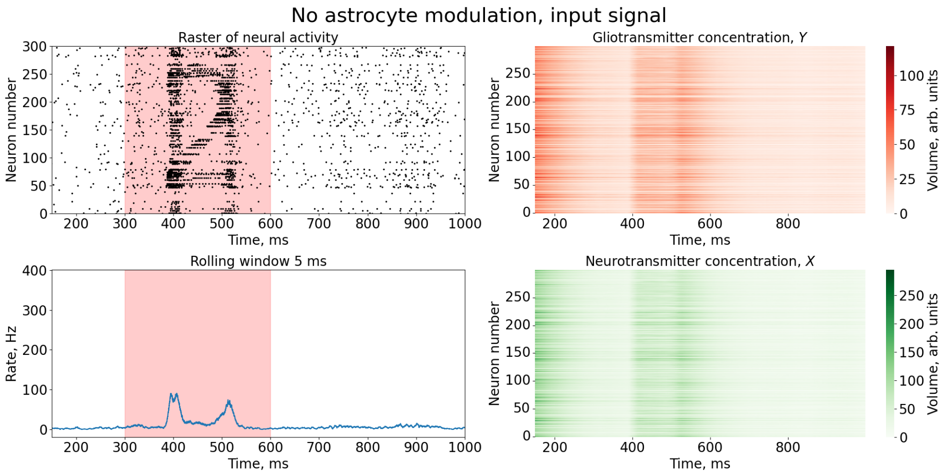

Figure S1. Time series of neural activity (upper left figure in the form of a raster diagram), the corresponding rate of activity of the neural network is the sum of the spikes of the neurons of the network, calculated on the time window 5 ms (lower left figure) and time series of concentrations gliotransmitters,Y, (upper right figure) and concentration neurotransmitters,X, (lower right figure). The red area in the figure indicates the timing of the input stimulus.

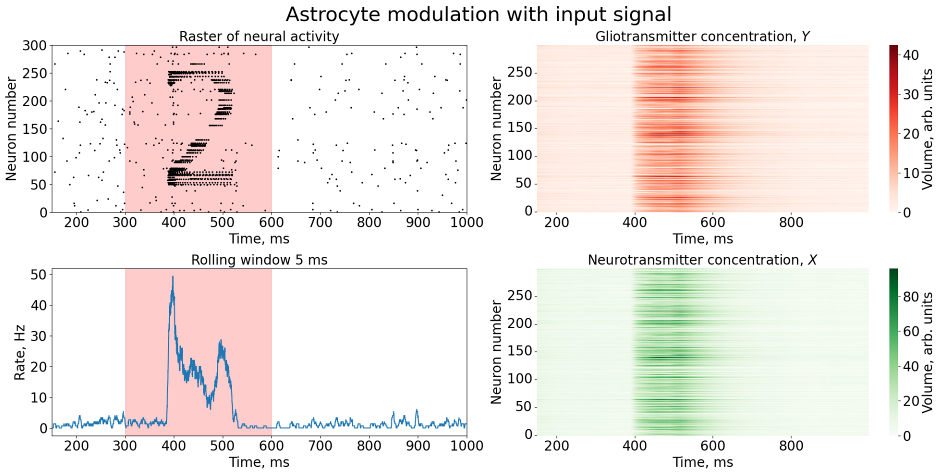

Figure S2. Time series of neural activity (upper left figure in the form of a raster diagram), the corresponding rate of activity of the neural network is the sum of the spikes of the neurons of the network, calculated on the time window 5 ms (lower left figure) and time series of concentrations gliotransmitters,Y, (upper right figure) and concentration neurotransmitters,X, (lower right figure). The red area in the figure indicates the timing of the input stimulus.

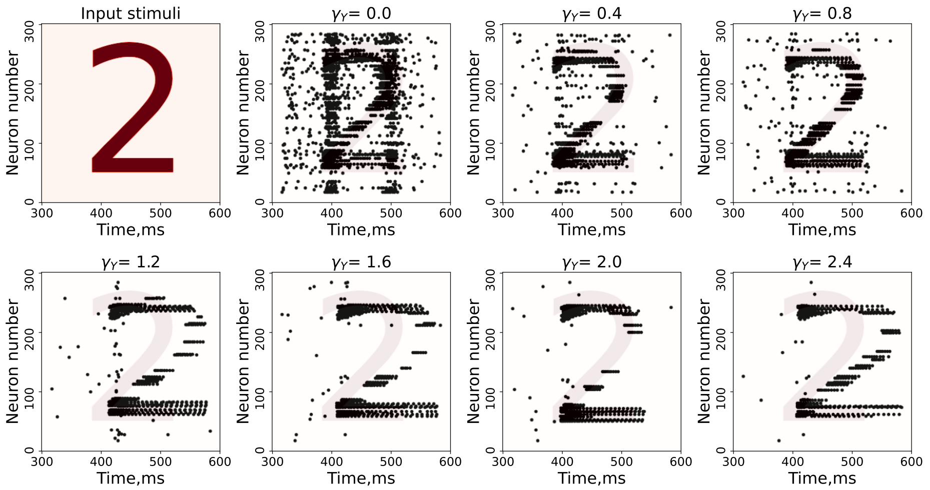

Figure S3. Part of the raster diagram of neural activity, demonstrating the representation of the supplied pattern at different parameter values γ_Y.

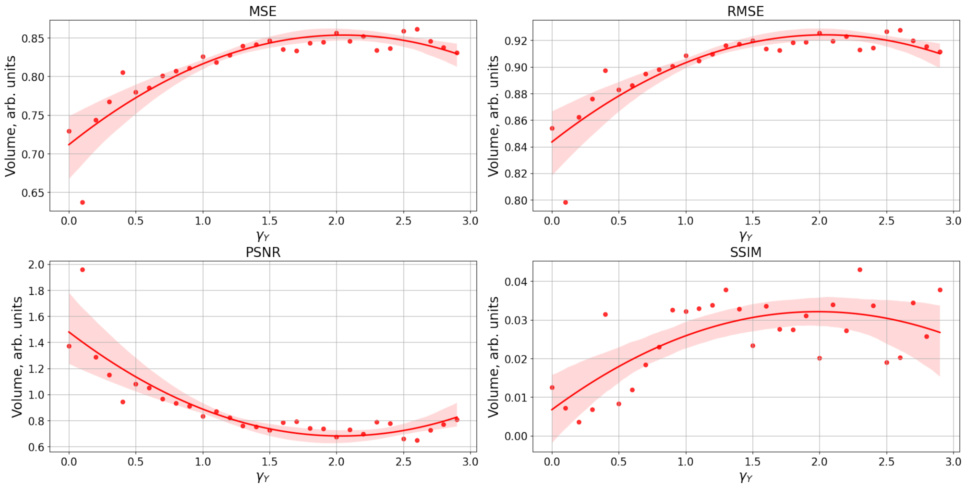

Figure S4. The dependence of the image comparison metrics (MSE, RMSE, PSNR and SSIM) for comparing the applied stimulus and the reproduced pattern by the neural network on the parameter value γ_Y. MSE - mean square error. RMSE - root mean square error. PSNR - peak signal to noise ratio. SSIM - structural similarity method.

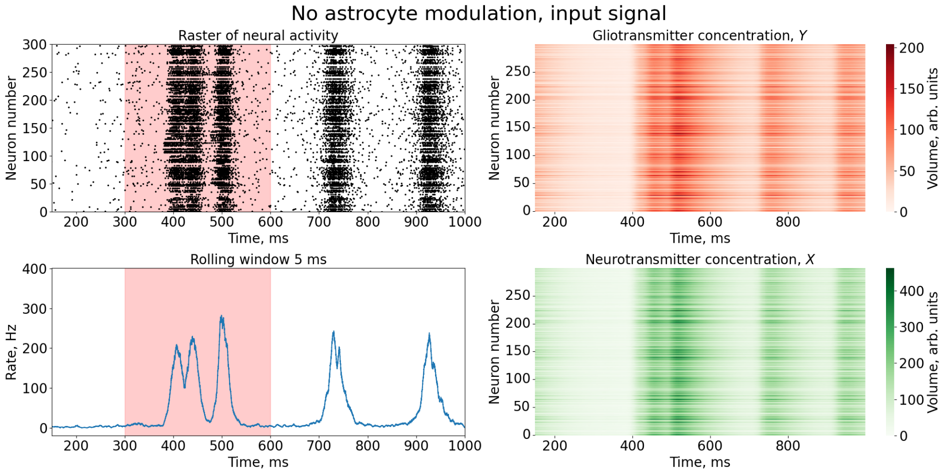

Figure S5. Time series of neural activity (upper left figure in the form of a raster diagram), the corresponding rate of activity of the neural network is the sum of the spikes of the neurons of the network, calculated on the time window 5 ms (lower left figure) and time series of concentrations gliotransmitters,Y, (upper right figure) and concentration neurotransmitters,X, (lower right figure). The red area in the figure indicates the timing of the input stimulus.

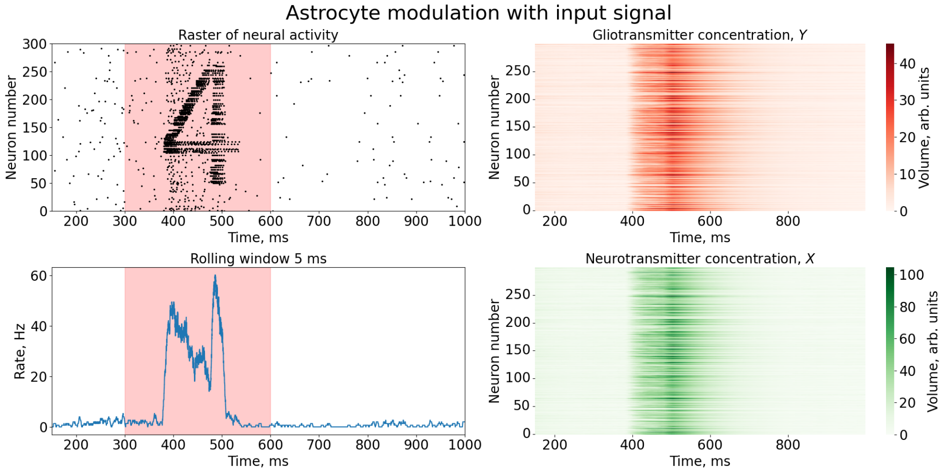

Figure S6. Time series of neural activity (upper left figure in the form of a raster diagram), the corresponding rate of activity of the neural network is the sum of the spikes of the neurons of the network, calculated on the time window 5 ms (lower left figure) and time series of concentrations gliotransmitters,Y, (upper right figure) and concentration neurotransmitters,X, (lower right figure). The red area in the figure indicates the timing of the input stimulus.

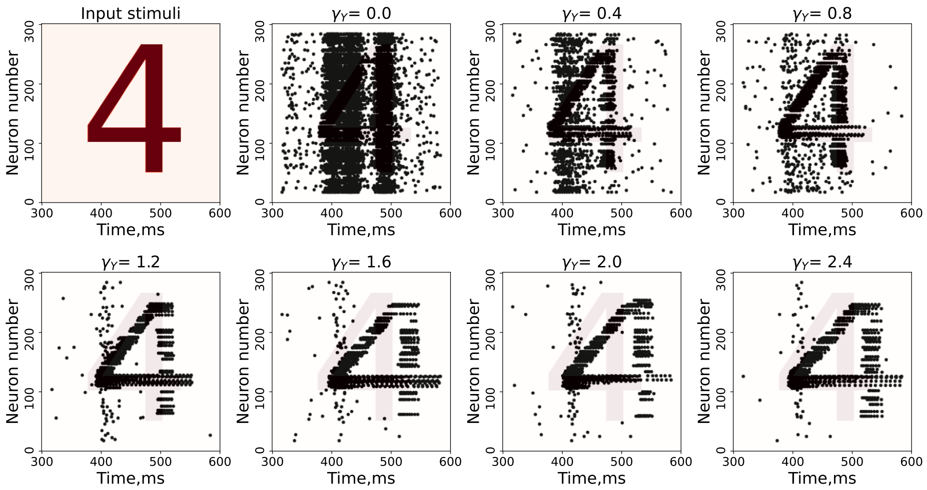

Figure S7. Part of the raster diagram of neural activity, demonstrating the representation of the supplied pattern at different parameter values γ_Y.

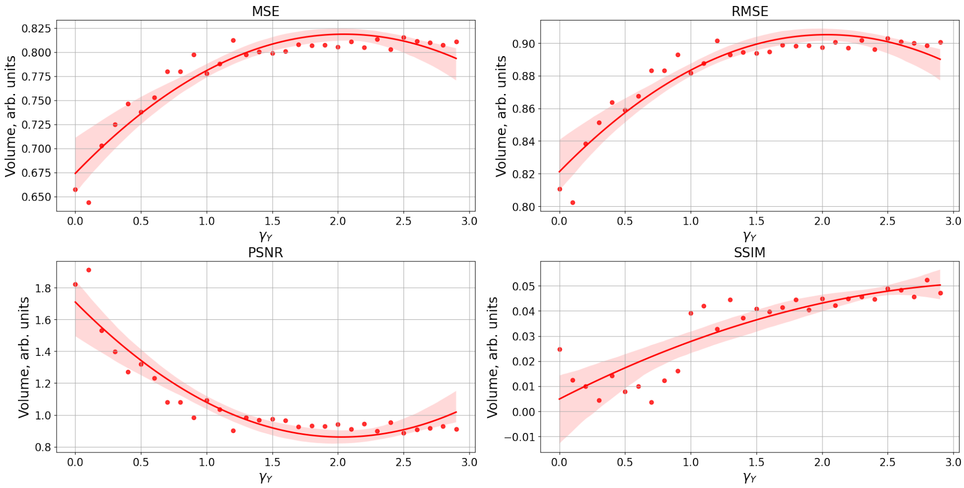

Figure S8. The dependence of the image comparison metrics (MSE, RMSE, PSNR and SSIM) for comparing the applied stimulus and the reproduced pattern by the neural network on the parameter value γ_Y. MSE - mean square error. RMSE - root mean square error. PSNR - peak signal to noise ratio. SSIM - structural similarity method.

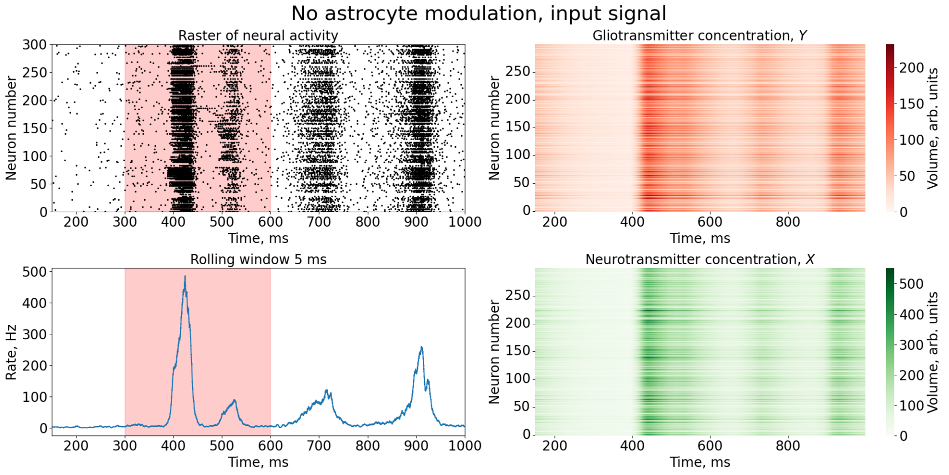

Figure S9. Time series of neural activity (upper left figure in the form of a raster diagram), the corresponding rate of activity of the neural network is the sum of the spikes of the neurons of the network, calculated on the time window 5 ms (lower left figure) and time series of concentrations gliotransmitters,Y, (upper right figure) and concentration neurotransmitters,X, (lower right figure). The red area in the figure indicates the timing of the input stimulus.

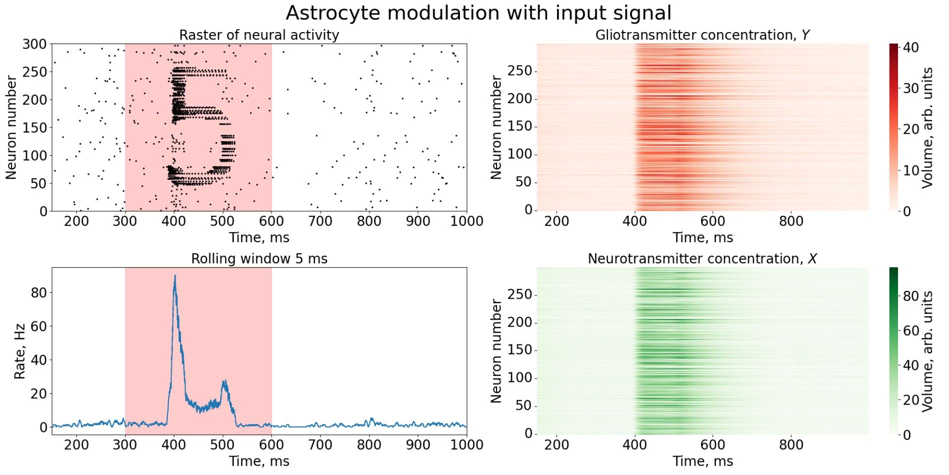

Figure S10. Time series of neural activity (upper left figure in the form of a raster diagram), the corresponding rate of activity of the neural network is the sum of the spikes of the neurons of the network, calculated on the time window 5 ms (lower left figure) and time series of concentrations gliotransmitters,Y, (upper right figure) and concentration neurotransmitters,X, (lower right figure). The red area in the figure indicates the timing of the input stimulus.

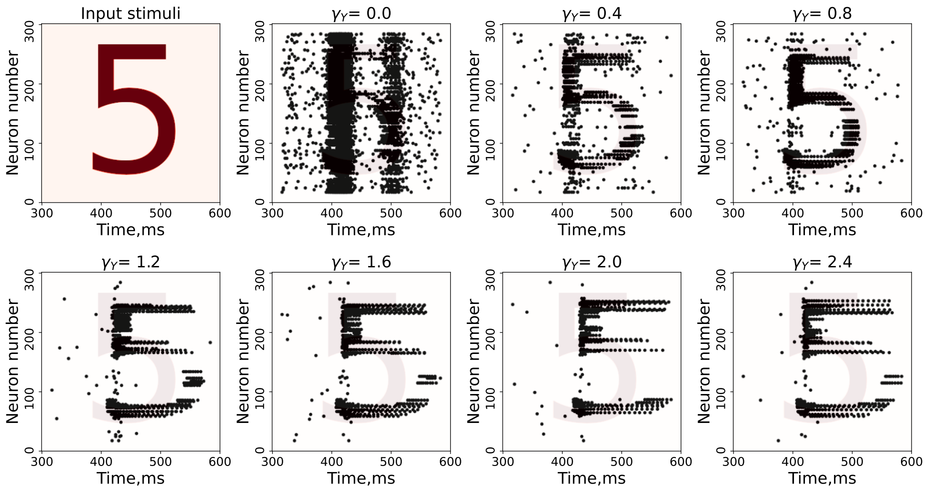

Figure S11. Part of the raster diagram of neural activity, demonstrating the representation of the supplied pattern at different parameter values γ_Y.

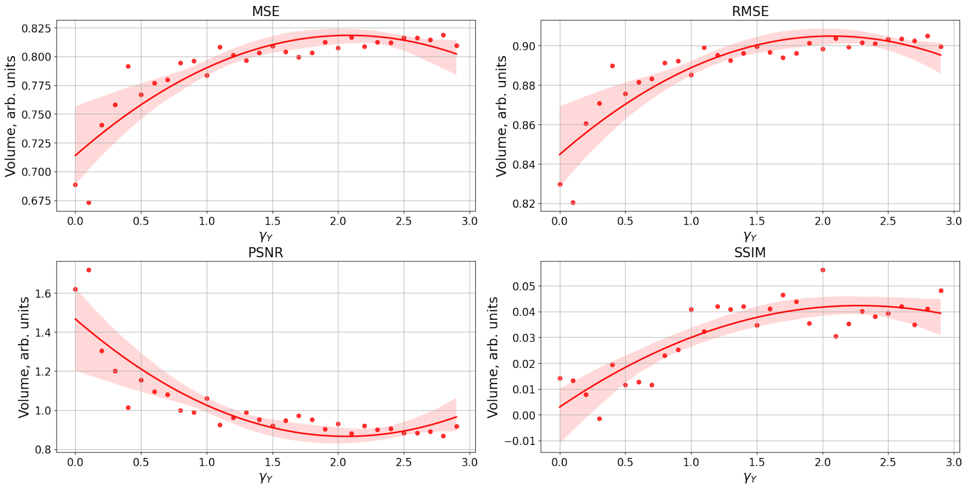

Figure S12. The dependence of the image comparison metrics (MSE, RMSE, PSNR and SSIM) for comparing the applied stimulus and the reproduced pattern by the neural network on the parameter value γ_Y. MSE - mean square error. RMSE - root mean square error. PSNR - peak signal to noise ratio. SSIM - structural similarity method.

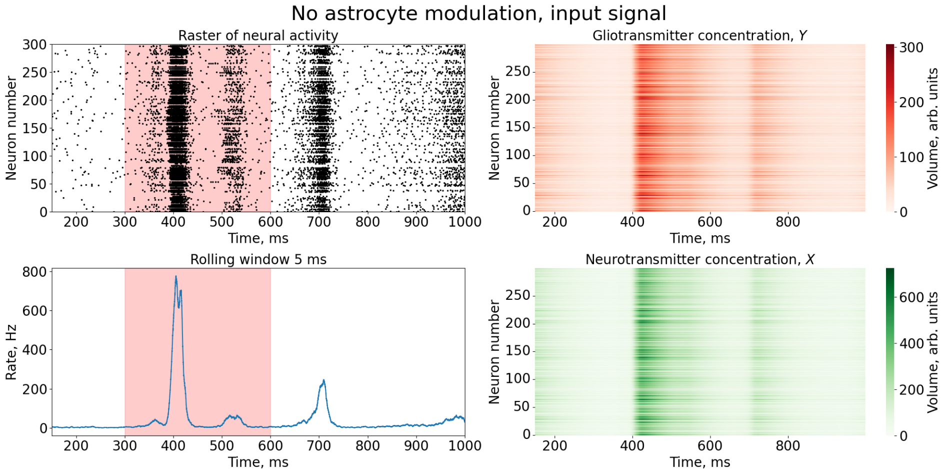

Figure S13. Time series of neural activity (upper left figure in the form of a raster diagram), the corresponding rate of activity of the neural network is the sum of the spikes of the neurons of the network, calculated on the time window 5 ms (lower left figure) and time series of concentrations gliotransmitters,Y, (upper right figure) and concentration neurotransmitters,X, (lower right figure). The red area in the figure indicates the timing of the input stimulus.

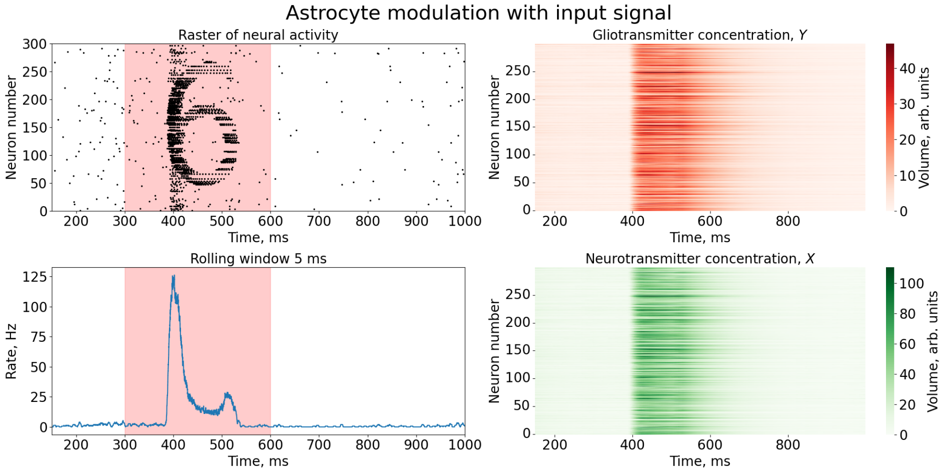

Figure S14. Time series of neural activity (upper left figure in the form of a raster diagram), the corresponding rate of activity of the neural network is the sum of the spikes of the neurons of the network, calculated on the time window 5 ms (lower left figure) and time series of concentrations gliotransmitters,Y, (upper right figure) and concentration neurotransmitters,X, (lower right figure). The red area in the figure indicates the timing of the input stimulus.

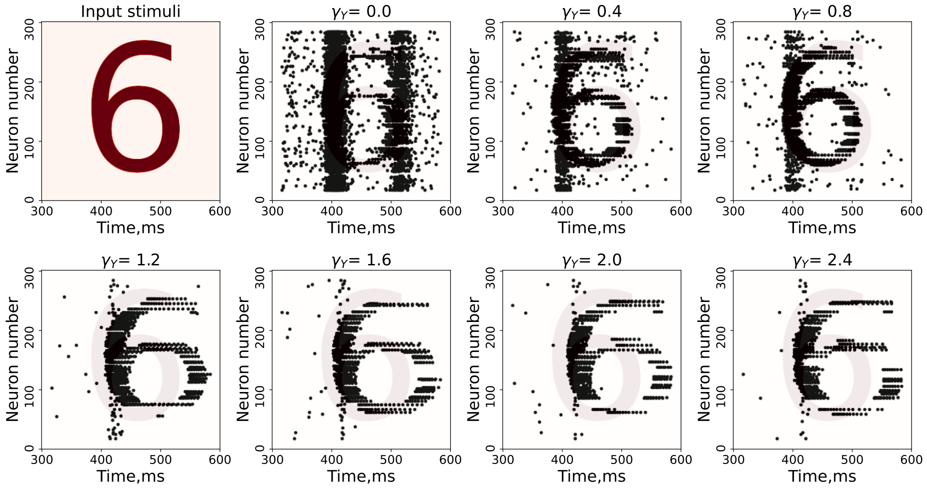

Figure S15. Part of the raster diagram of neural activity, demonstrating the representation of the supplied pattern at different parameter values γ_Y.

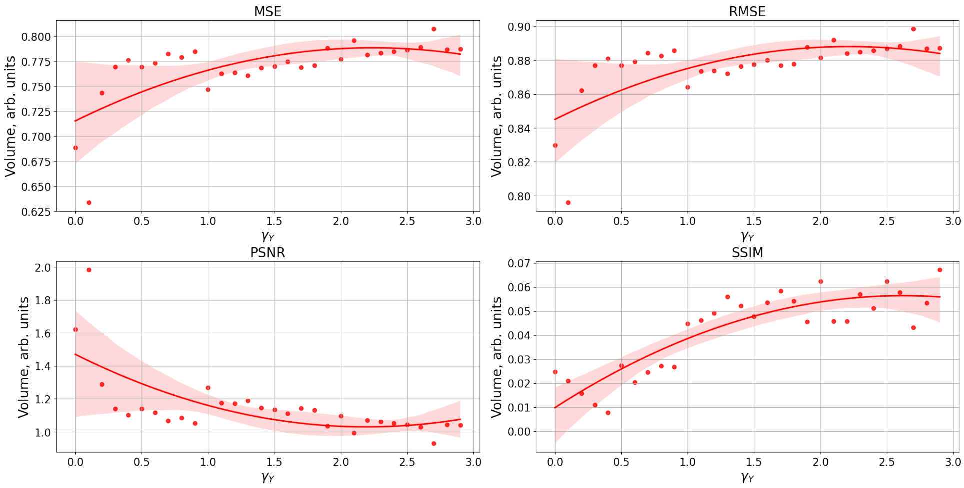

Figure S16. The dependence of the image comparison metrics (MSE, RMSE, PSNR and SSIM) for comparing the applied stimulus and the reproduced pattern by the neural network on the parameter value γ_Y. MSE - mean square error. RMSE - root mean square error. PSNR - peak signal to noise ratio. SSIM - structural similarity method.

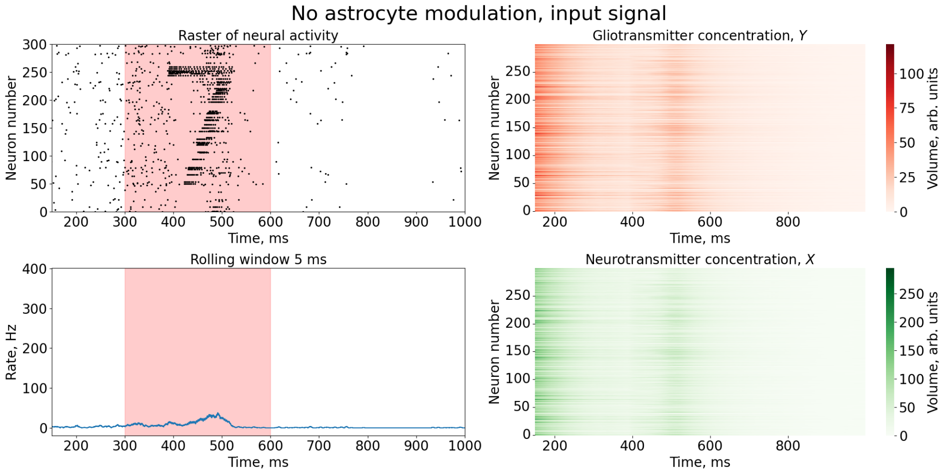

Figure S17. Time series of neural activity (upper left figure in the form of a raster diagram), the corresponding rate of activity of the neural network is the sum of the spikes of the neurons of the network, calculated on the time window 5 ms (lower left figure) and time series of concentrations gliotransmitters,Y, (upper right figure) and concentration neurotransmitters,X, (lower right figure). The red area in the figure indicates the timing of the input stimulus.

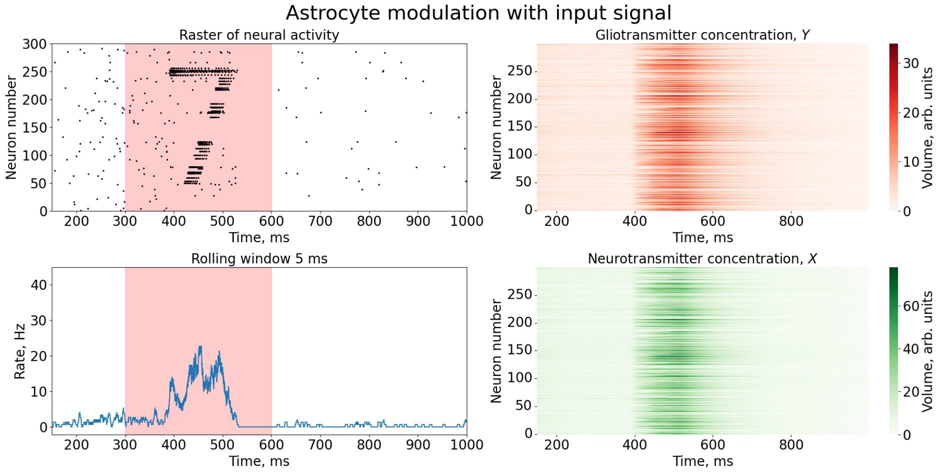

Figure S18. Time series of neural activity (upper left figure in the form of a raster diagram), the corresponding rate of activity of the neural network is the sum of the spikes of the neurons of the network, calculated on the time window 5 ms (lower left figure) and time series of concentrations gliotransmitters,Y, (upper right figure) and concentration neurotransmitters,X, (lower right figure). The red area in the figure indicates the timing of the input stimulus.

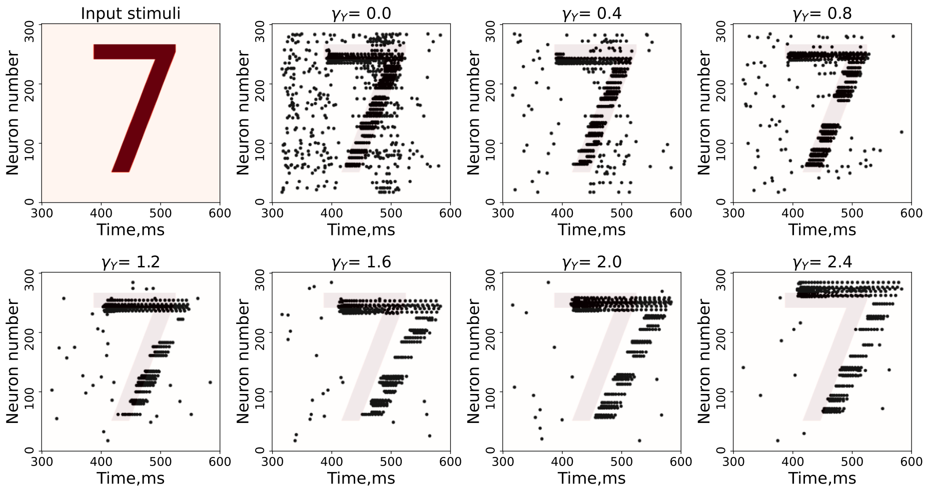

Figure S19. Part of the raster diagram of neural activity, demonstrating the representation of the supplied pattern at different parameter values γ_Y.

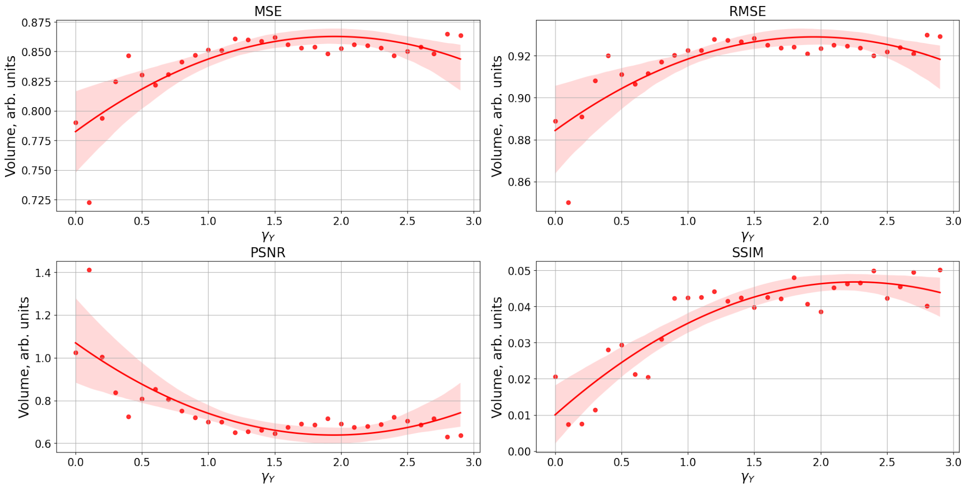

Figure S20. The dependence of the image comparison metrics (MSE, RMSE, PSNR and SSIM) for comparing the applied stimulus and the reproduced pattern by the neural network on the parameter value γ_Y. MSE - mean square error. RMSE - root mean square error. PSNR - peak signal to noise ratio. SSIM - structural similarity method.

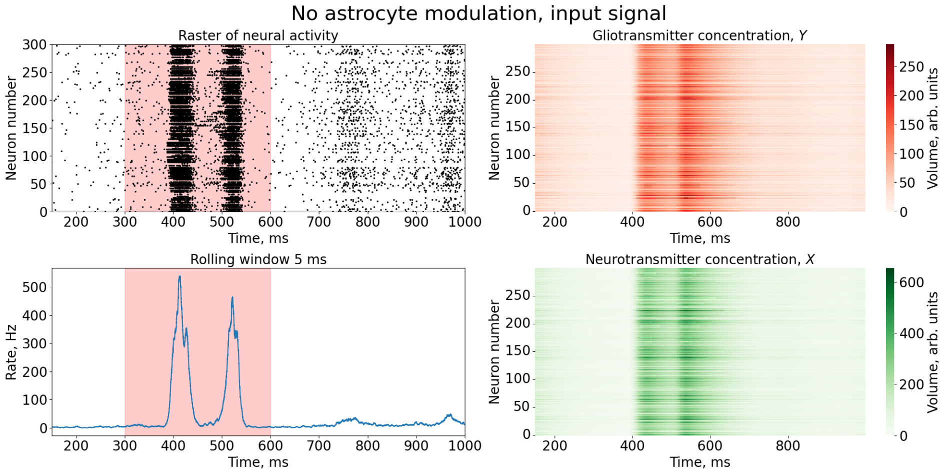

Figure S21. Time series of neural activity (upper left figure in the form of a raster diagram), the corresponding rate of activity of the neural network is the sum of the spikes of the neurons of the network, calculated on the time window 5 ms (lower left figure) and time series of concentrations gliotransmitters,Y, (upper right figure) and concentration neurotransmitters,X, (lower right figure). The red area in the figure indicates the timing of the input stimulus.

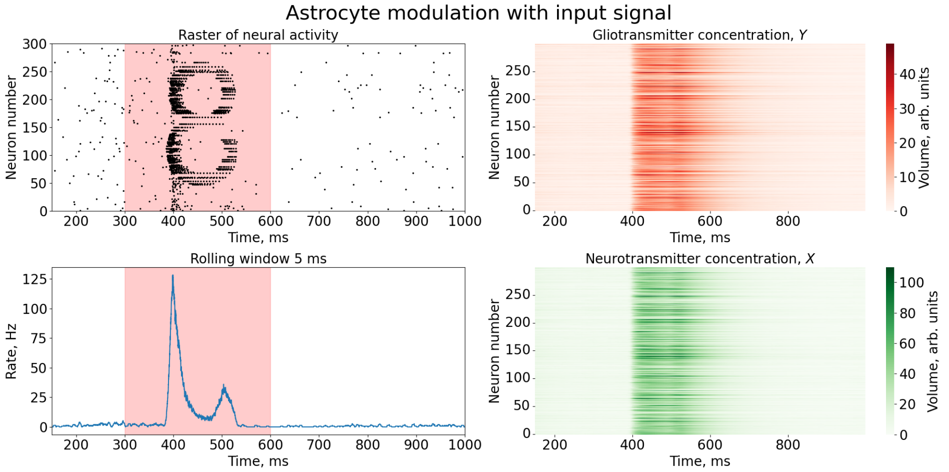

Figure S22. Time series of neural activity (upper left figure in the form of a raster diagram), the corresponding rate of activity of the neural network is the sum of the spikes of the neurons of the network, calculated on the time window 5 ms (lower left figure) and time series of concentrations gliotransmitters,Y, (upper right figure) and concentration neurotransmitters,X, (lower right figure). The red area in the figure indicates the timing of the input stimulus.

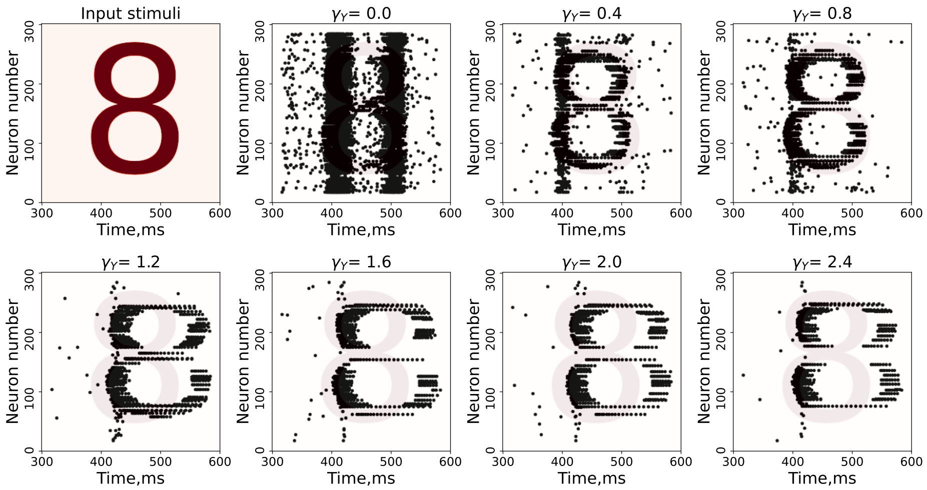

Figure S23. Part of the raster diagram of neural activity, demonstrating the representation of the supplied pattern at different parameter values γ_Y.

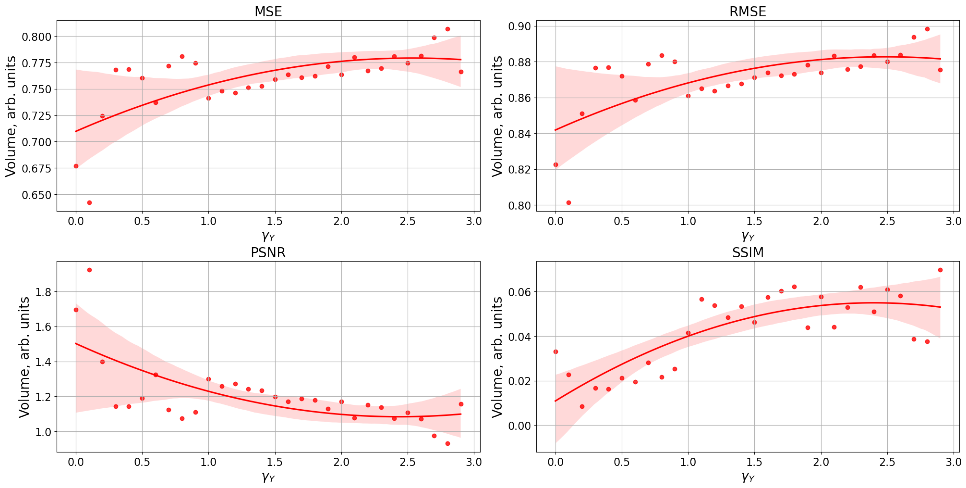

Figure S24. The dependence of the image comparison metrics (MSE, RMSE, PSNR and SSIM) for comparing the applied stimulus and the reproduced pattern by the neural network on the parameter value γ_Y. MSE - mean square error. RMSE - root mean square error. PSNR - peak signal to noise ratio. SSIM - structural similarity method.

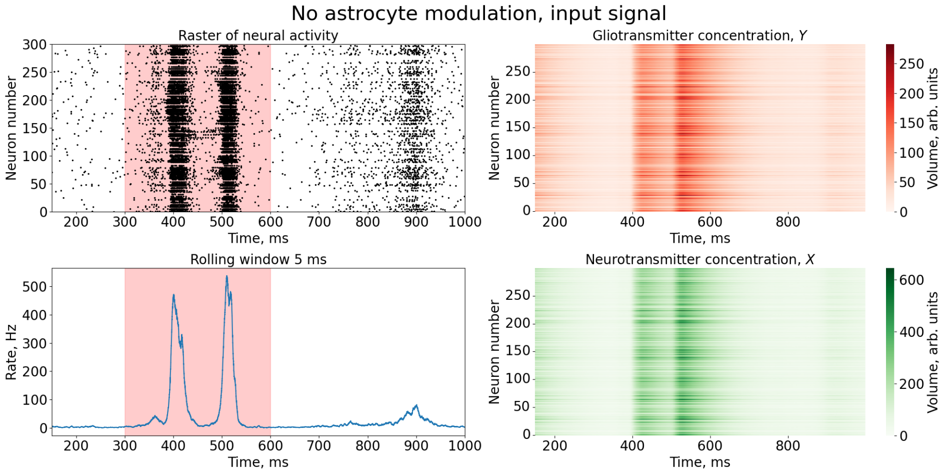

Figure S25. Time series of neural activity (upper left figure in the form of a raster diagram), the corresponding rate of activity of the neural network is the sum of the spikes of the neurons of the network, calculated on the time window 5 ms (lower left figure) and time series of concentrations gliotransmitters,Y, (upper right figure) and concentration neurotransmitters,X, (lower right figure). The red area in the figure indicates the timing of the input stimulus.

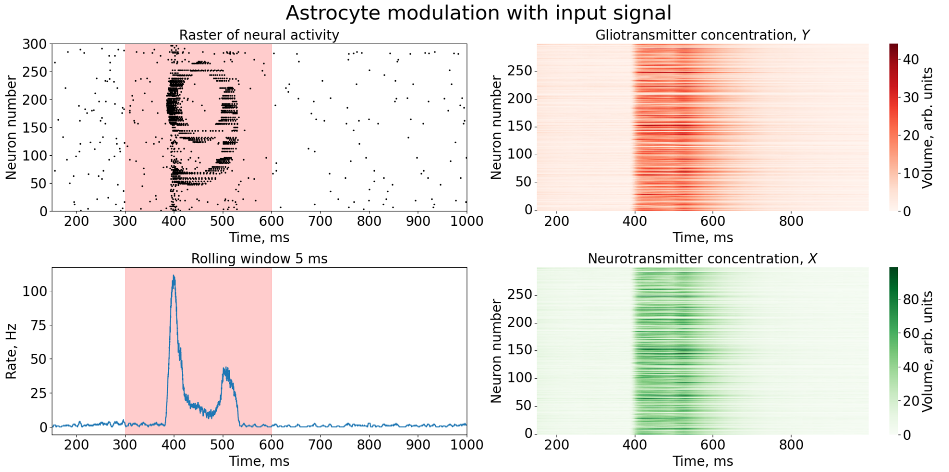

Figure S26. Time series of neural activity (upper left figure in the form of a raster diagram), the corresponding rate of activity of the neural network is the sum of the spikes of the neurons of the network, calculated on the time window 5 ms (lower left figure) and time series of concentrations gliotransmitters,Y, (upper right figure) and concentration neurotransmitters,X, (lower right figure). The red area in the figure indicates the timing of the input stimulus.

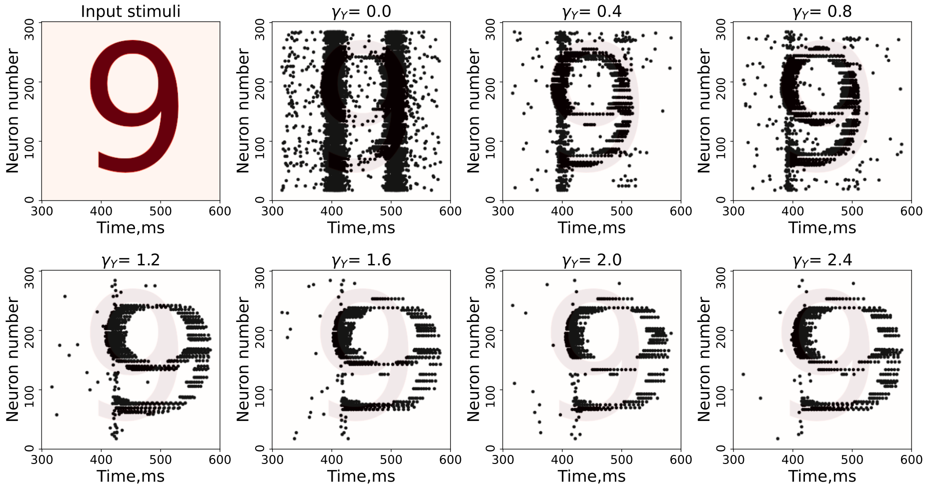

Figure S27. Part of the raster diagram of neural activity, demonstrating the representation of the supplied pattern at different parameter values γ_Y.

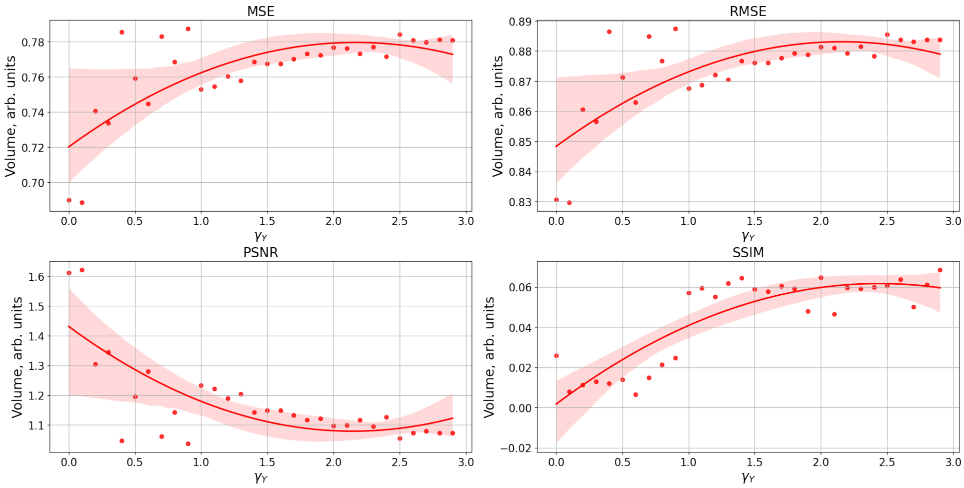

Figure S28. Dependence of the image comparison metrics (MSE, RMSE, PSNR and SSIM) of the task of comparing the applied stimulus and the reproduced pattern by the neural network on the parameter value γ_Y. MSE - mean square error. RMSE - root mean square error. PSNR - peak signal to noise ratio. SSIM - structural similarity method.
